# Supplementary material for: Ranking lifestyle risk factors for cervical cancer among Black women: A case-control study from Johannesburg, South Africa
Source: PLoS One. 2021 Dec 8;16(12):e0260319. doi: 10.1371/journal.pone.0260319 (PMC8654217; doi:10.1371/journal.pone.0260319)
Supplement: S1 Table — (DOCX) [file pone.0260319.s001.docx]

S1 Table

| Cancer type | ICD-O-3-code | Frequency  N= 5,709 | Infection unrelated cancer controls | Smoking unrelated cancer controls | Alcohol unrelated cancer controls | Hormonal contraceptive unrelated cancer controls |
| --- | --- | --- | --- | --- | --- | --- |
| Breast | C50 | 3,843 |  |  |  |  |
| Colon | C18-20 | 340 |  |  |  |  |
| Endometrium | C54-55 | 224 |  |  |  |  |
| Lung Cancer | C33-34 | 159 |  |  |  |  |
| Myeloid Leukaemia | ICD-10 C92 | 105 |  |  |  |  |
| Myeloma | C90 | 123 |  |  |  |  |
| Oesophagus | C15 | 314 |  |  |  |  |
| Ovaries | C56 | 263 |  |  |  |  |
| Pancreas | C25 | 51 |  |  |  |  |
| minor cancers |  |  |  |  |  |  |
| Melanoma | C43 | 39 |  |  |  |  |
| Larynx | C32 | 27 |  |  |  |  |
| Kidney | C64 | 20 |  |  |  |  |
| Fallopian tube | C57.0 | 31 |  |  |  |  |
| Meninges | C70 | 1 |  |  |  |  |
| Endocrine gland | C75 | 2 |  |  |  |  |
| Bone | C40-41 | 33 |  |  |  |  |
| Brain | C71 | 14 |  |  |  |  |
| Central nervous system (CNS) | C72 | 1 |  |  |  |  |
| Peritoneum and retroperitoneum | C48 | 9 |  |  |  |  |
| Placenta | C58.9 | 33 |  |  |  |  |
| Small Intestine | C17 | 9 |  |  |  |  |
| Soft Tissue Sarcoma | C49 | 26 |  |  |  |  |
| Thymus | C37 | 5 |  |  |  |  |
| Thyroid | C73 | 37 |  |  |  |  |
