# Supplementary material for: Ranking lifestyle risk factors for cervical cancer among Black women: A case-control study from Johannesburg, South Africa
Source: PLoS One. 2021 Dec 8;16(12):e0260319. doi: 10.1371/journal.pone.0260319 (PMC8654217; doi:10.1371/journal.pone.0260319)
Supplement: Attachment — Submitted filename: Response to the reviewers.docx [file pone.0260319.s003.docx]

1. Please ensure that your manuscript meets PLOS ONE's style requirements, including those for file naming. The PLOS ONE style templates can be found at

<https://journals.plos.org/plosone/s/file?id=wjVg/PLOSOne_formatting_sample_main_body.pdf> and

<https://journals.plos.org/plosone/s/file?id=ba62/PLOSOne_formatting_sample_title_authors_affiliations.pdf>.

Response :

**Thank you for your comment: The manuscript has been formatted and meets PLOSE ONE’s style requirement.**

2. Please include additional information regarding the survey or questionnaire used in the study and ensure that you have provided sufficient details that others could replicate the analyses. For instance, if you developed the survey or questionnaire as part of this study and it is not under a copyright more restrictive than CC-BY, please include a copy, in both the original language and English, as Supporting Information. If the questionnaire is published, please provide a citation to the (1) questionnaire and/or (2) original publication associated with the questionnaire.

Response:

**Thank you for your comment: Additional information regarding the questionnaire has been added to the manuscript line 141-147. Page 5, as follows:**

The questionnaire included questions on the following: socio-demographic factors such as; place of birth and residence, marital status, education, the home language of parents. Enviromental exposures such as method of cooking and heating. Lifestyle factors such as; smoking by type of tobacco and amounts smoked, snuff (sniffed tobacco) use, alcohol consumption by type, parity, use of oral and injectable contraceptives, number of sexual partners. On occupations, self-reported use of Anti-Retroviral Therapy (ART) (since 2005), PAP smear (2001) and self-reported history of diabetes.

**The questionnaire has also been included as a Supplementary material.**

3. In your ethics statement in the Methods section and in the online submission form, please provide additional information about the data used in your retrospective study. Specifically, please ensure that you have discussed whether all data were fully anonymized before you accessed them and/or whether the IRB or ethics committee waived the requirement for informed consent. If patients provided informed written consent to have data from their medical records used in research, please include this information.

Response:

**Thank you for your comment: Information on data anonymization has been discussed in the revised manuscript lines 147-152 page 5 as follows:**

The JCS and the current study were approved by the University of the Witwatersrand Human Research Ethics Committee (Medical) (certificate number for the current study. M200252). In the JCS, participants gave written informed or wittnessd consent to once-off interview and optional blood draw and to have their information and blood sample anonymized. Any future investigations require approval of the University of the Witwatersrand Human Research Ethics Committee HREC [16].

**The information has also been included in the online submission form.**

4. Please include your actual numerical p-values in Table 2.

**Response:**

**Thank you for your comment: The actual numerical p-values in Table 2 have been added. Pages 13-15**

5. In your Data Availability statement, you have not specified where the minimal data set underlying the results described in your manuscript can be found. PLOS defines a study's minimal data set as the underlying data used to reach the conclusions drawn in the manuscript and any additional data required to replicate the reported study findings in their entirety. All PLOS journals require that the minimal data set be made fully available. For more information about our data policy, please see <http://journals.plos.org/plosone/s/data-availability>.

**Response:**

**Data cannot be shared publicly because of ethics policy at University of Witwatersrand, whereby any new analyses require Human Research Ethics Committee approval. Data are available from the SA-NCR /National Health Laboratory Services. (contact :** [**elviras@nicd.ac.za**](mailto:elviras@nicd.ac.za)**) for researchers who meet the relevant ethics criteria for access to these data.**

**Reviewer 1:**

Although the manuscript is well statistics, according to previous references, all information that we had known with the lifestyle risk factors for cervical cancer among Black women, like these related references that authors cited, so for more contribution, suggest authors clarify and mentation what these JCS data that the medical system or policy-related cervical cancer could improve. Thank you for your effort.

**Response:**

**Thank you very much for your question:**

**Our findings have important policy implications. Firstly, ranking of lifestyle risk factors for cervical cancer can inform medical systems on which key risk factors to integrate in cervical cancer education programmes. This would help health care personnel that are involved in advising women about cervical cancer screening, to understand the public-health gains that result by minimizing the risk of each factor. Second, most of the previous studies were conducted in women of European ancestry. We have amended the concluding sentence as follows:**

In conclusion**,** in order of importance, HIV-positivity, educational attainment, parity, hormonal contraceptive use, alcohol, smoking and residing in rural area were associated with cervical cancer among black South African women. These women should be prioritized in opportunistic or planned cervical cancer screening programs. Our findings confirm previosuly known cofactors of cervical cancer and provide a rank order of risks that could be used locally to target educational messaging and appropriate interventions.

Reviewer #2:

This is a good study to determine the risk factors for cervical cancer, one of the most common cancer types in women.It is a case-control study on a large sample that will contribute to the literature.

**We thank the reviewer for their comments.**
