# Supplementary material for: Ranking lifestyle risk factors for cervical cancer among Black women: A case-control study from Johannesburg, South Africa
Source: PLoS One. 2021 Dec 8;16(12):e0260319. doi: 10.1371/journal.pone.0260319 (PMC8654217; doi:10.1371/journal.pone.0260319)
Supplement: Attachment — Submitted filename: Response to the reviewers_25 October 2021.docx [file pone.0260319.s004.docx]

**Journal Requirements:**

Please review your reference list to ensure that it is complete and correct.

**Response:**

**Reference list has been reviewed, it is complete and correct**

**Reviewer #1: Dear authors,**
The revised manuscript is better, but Result section, “A total of, 26,263 participants were enrolled in the JCS between 1995 to 2016. A total of 9,249 women were included in this study of whom 3,540 (38.3%) had invasive cervical cancer (cases) and 5,709 were designated as controls, (61.7%) as described in Figure 1.”The figure is vague, not easy to read follow the authors’ context, please clarify.
Thank you.

**Response:**

**Thank you so much for your question:**

**Figure 1 has been revised for easy to read. The information on the results section regarding figure 1, has been placed in the methods section where the description of exclusion was done. (lines 161-184 of the manuscript).**

A total of, 26,263 participants were enrolled in the JCS between 1995 to 2016. We excluded 8,677 (33.0%) males, 3,105 (17.6%) women older than 65 years or younger than 25 years, 663 (4.6%) non-cancer participants, 945(6.9%) non-South Africans, 1 with missing data, 640 (5.0%) with missing HIV-status and 691 (5.7%) with primary site unknown malignancy. From those with cancer of the cervix, we excluded International Classification of Diseases for Oncology (ICD-O) codes such as ( ICD-O morphology: 8010-8050 epithelial neoplasm (n=98, 2.6%), (ICD-O morphology: 8000 and 8001) not otherwise specified (n=9, 0.2%), (ICD-O morphology: 8560, 8570 and 8574) complex epithelial neoplasms (n=108, 2.8%) and other minor histological types (ICD-O morphology: 8098,8170, 8200, 8272, 8310, 8441,8460, 8480-8490, 8500,8650 and 8931-9100) (n=64, 1.7%) Figure 1.

Figure 1 also outlines the criteria used to select cases and controls. The control groups were arranged into four sets. Each set comprised of women diagnosed with different cancer types that are unrelated to the exposures of interest (these being infection, smoking, alcohol, parity, number of sexual partners and use of hormonal contraceptives) [16–18] Figure 1. Cancer controls unrelated to infection constituted the highest number of controls S1 Table. The demographic analyses excluded 2,009 (26.0%) women with cancers related to infections. Analyses relating to smoking excluded 2,218 (28.7%) women with cancers related to smoking and infections. Analyses relating to alcohol excluded 6,800 (60.4%) women with cancers related to alcohol and infections. Analyses relating to hormonal contraceptives excluded 6,458 (57.4%) women with cancers related to hormonal contraceptives and infections. Of the remaining 9,249 women, 3,540 (38.3%) had invasive cervical cancer (defined as cases) and 5,709 (61.7%) had other cancers (defined as controls) that were not related to the exposure of interest Figure1.

**Reviewer #2:**

An original study done in a special group. It is a good work that will contribute to the literature.

**We thank the reviewer for their comments.**
